# Comparison of triglyceride glucose index and modified triglyceride glucose indices in prediction of cardiovascular diseases in middle aged and older Chinese adults

**DOI:** 10.1186/s12933-024-02278-z

**Published:** 2024-05-29

**Authors:** Cancan Cui, Yitian Qi, Jiayin Song, Xinyun Shang, Tianjiao Han, Ning Han, Siqi Yue, Yining Zha, Zhonghang Xu, Jiannan Li, Lin Liu

**Affiliations:** 1 China-Japan Union Hospital of Jilin University, Jilin University, Jilin, China; 2 Harvard T H Chan School of Public Health, Boston, USA

**Keywords:** Triglyceride and glucose index, Modified TyG indices, Cardiovascular disease, Time-dependent ROC analysis

## Abstract

**Background:**

Triglyceride and glucose (TyG) index, a surrogate marker of insulin resistance, has been validated as a predictor of cardiovascular disease. However, effects of TyG-related indices combined with obesity markers on cardiovascular diseases remained unknown. We aimed to investigate the associations between TyG index and modified TyG indices with new-onset cardiovascular disease and the time-dependent predictive capacity using a national representative cohort.

**Methods:**

This study is a retrospective observational cohort study using data from China Health and Retirement Longitudinal Study (CHARLS) of 7 115 participants. The TyG index was calculated as Ln [fasting triglyceride (mg/dL) × fasting glucose (mg/dL)/2]. The modified TyG indices were developed combining TyG with body mass index (BMI), waist circumference (WC) and waist-to‐height ratio (WHtR). We used adjusted Cox proportional hazards regression to analyze the association and predictive capacity based on hazard ratio (HR) and Harrell’s C‐index.

**Results:**

Over a 7-year follow‐up period, 2136 participants developed cardiovascular disease, including 1633 cases of coronary heart disease and 719 cases of stroke. Compared with the lowest tertile group, the adjusted HR (95% CI) for new-onset cardiovascular disease in the highest tertile for TyG, TyG-BMI, TyG-WC, and TyG-WHtR were 1.215 (1.088–1.356), 1.073 (0.967–1.191), 1.078 (0.970–1.198), and 1.112 (1.002–1.235), respectively. The C‐indices of TyG index for cardiovascular disease onset were higher than other modified TyG indices. Similar results were observed for coronary heart disease and stroke.

**Conclusion:**

TyG and TyG-WhtR were significantly associated with new-onset cardiovascular diseases, and TyG outperformed the modified TyG indices to identify individuals at risk of incident cardiovascular event.

**Supplementary Information:**

The online version contains supplementary material available at 10.1186/s12933-024-02278-z.

## Background

Insulin resistance plays a key role in the development of diabetes and atherosclerotic cardiovascular diseases [1, 2]. Using insulin resistance assessment to evaluate cardiovascular risk is of particular importance in the general population [3]. In a clinical setting, insulin resistance is commonly evaluated using hyperinsulinemic-euglycemic clamp test and fasting insulin (e.g., HOMA-IR) [4]. In epidemiological studies, triglyceride-glucose (TyG) index, developed using fasting triglyceride and blood glucose, has been proposed as a simple and reliable surrogate marker of insulin resistance [5]. Previous studies have recognized that TyG index is independently associated with atherosclerosis and cardiovascular disease, such as hypertension [6], carotid plaque [7], coronary heart disease and stroke [8].

Recently, modified TyG indices combining TyG and body composition indices, such as body mass index (BMI), waist circumference (WC) and waist-to-height ratio, have been developed to track the risk of metabolic diseases. TyG-WC is a novel and effective predictor of diabetes in first-degree relatives of type 2 diabetes patients [9]. TyG index combined with BMI, WC, and WHtR can further stratify and predict the risk of diabetes [10]. TyG and modified TyG indices are validated to identify individuals at risk of hypertension [11]. However, there is scare evidence examining the relationship between TyG and modified TyG indices with cardiovascular disease.

Using a national representative cohort, we aimed to investigate the association and the predictive capacity of TyG index and modified TyG indices with new-onset cardiovascular diseases in the middle aged and older Chinese population.

## Methods

### Study population

This current study was a secondary analysis using data from China Health and Retirement Longitudinal Study (CHARLS). CHARLS (http://charls.pku.edu.cn/) is a national project aiming to investigate the policy and health related data among adults aged 45 years or older and solve the population aging issue. There are four surveys between 2011 and 2018, with participants recruited from both rural and urban residence using multistage stratified probability proportional-to-size sampling strategy. Details of the study design and cohort profile have been previously described [12]. CHARLS data have been widely used in the epidemiolocal research [13–15]. The CHARLS study was approved by the Institutional Review Board of Peking University (IRB00001052-11015).

Following the study design of our previous study [8], the first survey (2011–2012) was set as baseline and participants were then followed at three subsequent visits (2013–2014, 2015–2016, 2017–2018). Participants with no history of cardiovascular diseases were primarily included in the current analysis. People lacking sociodemographic characteristics (age and sex), necessary blood tests and anthropometric measurements, or data of cardiovascular diseases or cancer history at baseline were further excluded. Then, a total of 7 115 participants were included in the final analysis.

### TyG and modified TyG indices

Plasma glucose (FPG) and triglyceride levels were analyzed using Hitachi 7180 chemistry analyzer (Hitachi, Tokyo, Japan). The coefficient of variation (CV) of blood marker measurement was < 5%. Height, weight, and waist circumference (WC) were physically measured three times, and the average values were adopted as the final measurements. BMI was calculated as weight (in kilograms)/height^2 (in meters squared). Waist-to-height ratio (WHtR) was defined as WC/height. TyG index was calculated as Ln [triglycerides (mg/dL) × glucose (mg/dL)/2]. TyG was modified by multiplying with BMI, WC, and WHtR to produce TyG-BMI, TyG-WC, and TyG-WHtR, respectively. TyG index and modified TyG indices were grouped into tertiles (T) as follows:

- TyG, T1: <8.36, T2: 8.36–8.87, and T3: >8.87.
- TyG-BMI, T1: <177.9, T2: 177.9-223.1, and T3: >223.1.
- TyG-WC, T1: <684.4, T2: 684.4-775.7, and T3: >775.7.
- TyG-WHtR, T1: <4.30, T2: 4.30–4.93, and T3: >4.93.

### New-onset cardiovascular events

The primary study outcome was the incidence of cardiovascular disease, a composite event of coronary heart disease and stroke. In accordance with previous studies [13, 16], new-onset cardiovascular events were assessed by the following standardized questions: “Have you been told by a doctor that you have been diagnosed with a heart attack, coronary heart disease, angina, congestive heart failure, or other heart problems?” or “Have you been told by a doctor that you have been diagnosed with a stroke?” Participants who reported heart disease or stroke during the follow-up period were defined as having new-onset cardiovascular disease. The outcomes were assessed by rigorously trained interviewers through standardized questionnaires that are harmonized to international leading aging surveys.

### Covariates

Information on socio-demographic status and health-related factors using a structured questionnaire was collected at baseline. Sociodemographic variables included age, sex, education (elementary school and below, secondary school, and college and above), marital status (married and others), and residence (rural, urban). Health-related factors included smoking and drinking status (yes or no), hypertension, diabetes, and use of medications for hypertension and diabetes. Smoking status was defined as “never smoking”, “current smoker” and “former smoker”. Subjects were diagnosed of hypertension when the systolic blood pressure was ≥ 140 mmHg or the diastolic pressure was ≥ 90 mmHg or self-reported diagnosis history of hypertension or antihypertensive medications currently used. Diabetes was defined as fasting glucose ≥ 7.0 mmol/L or self-reported diagnosis history of diabetes or use of any hypoglycemic medication currently used.

### Statistical analysis

Data are presented as means ± standard deviation (SD) for continuous variables and number (percentages) for categorical variables. Baseline characteristics were summarized based on TyG index tertile groups. The correlations of TyG, TyG-BMI, TyG-WC and TyG-WHtR are shown using Spearman’s coefficients.

The cumulative incidence rate of new-onset events based on tertile of TyG and modified TyG indices are presented as Kaplan-Meier curves. Multivariable-adjusted Cox regression models are used to investigate the association between TyG and modified TyG indices with cardiovascular disease onset. Hazard ratio (HR) with 95% confidence interval (CI) are calculated. The proportional hazards assumption is tested using Schoenfeld residuals, and no potential violation was observed. To account for potential confounders, we conducted the analysis in two models: age (continuous) and sex are adjusted in model 1, and in model 2, residence (rural, urban), education level (primary, secondary, third), marital status (married, others), smoking status (current, former, never), current drinking (yes, no), hypertension (yes, no) and diabetes (yes, no) are further adjusted. To illustrate the dose-response relationship of TyG and modified TyG indices, we performed the restricted cubic spline function analysis using 3 knots at the 10th, 50th, and 90th percentiles. The reference point is set as the median value of variables among the corresponding populations. In addition, the effects of TyG and modified TyG indices on coronary heart disease and stroke are analyzed.

We calculated the time-dependent Harrell’s C-index (95% CI) to evaluate the predictive capacity of TyG, TyG-BMI, TyG-WC, and TyG-WHtR for new-onset cardiovascular disease, coronary heart disease and stroke. Given potential sex difference, we performed the subgroup analysis in terms of sex. All statistical analyses were performed using R version 4.1.0 (R Foundation for Statistical Computing), and a two-sided P value < 0.05 was considered to indicate statistical significance.

## Results

### Characteristics of the study population

**Fig. 1:**
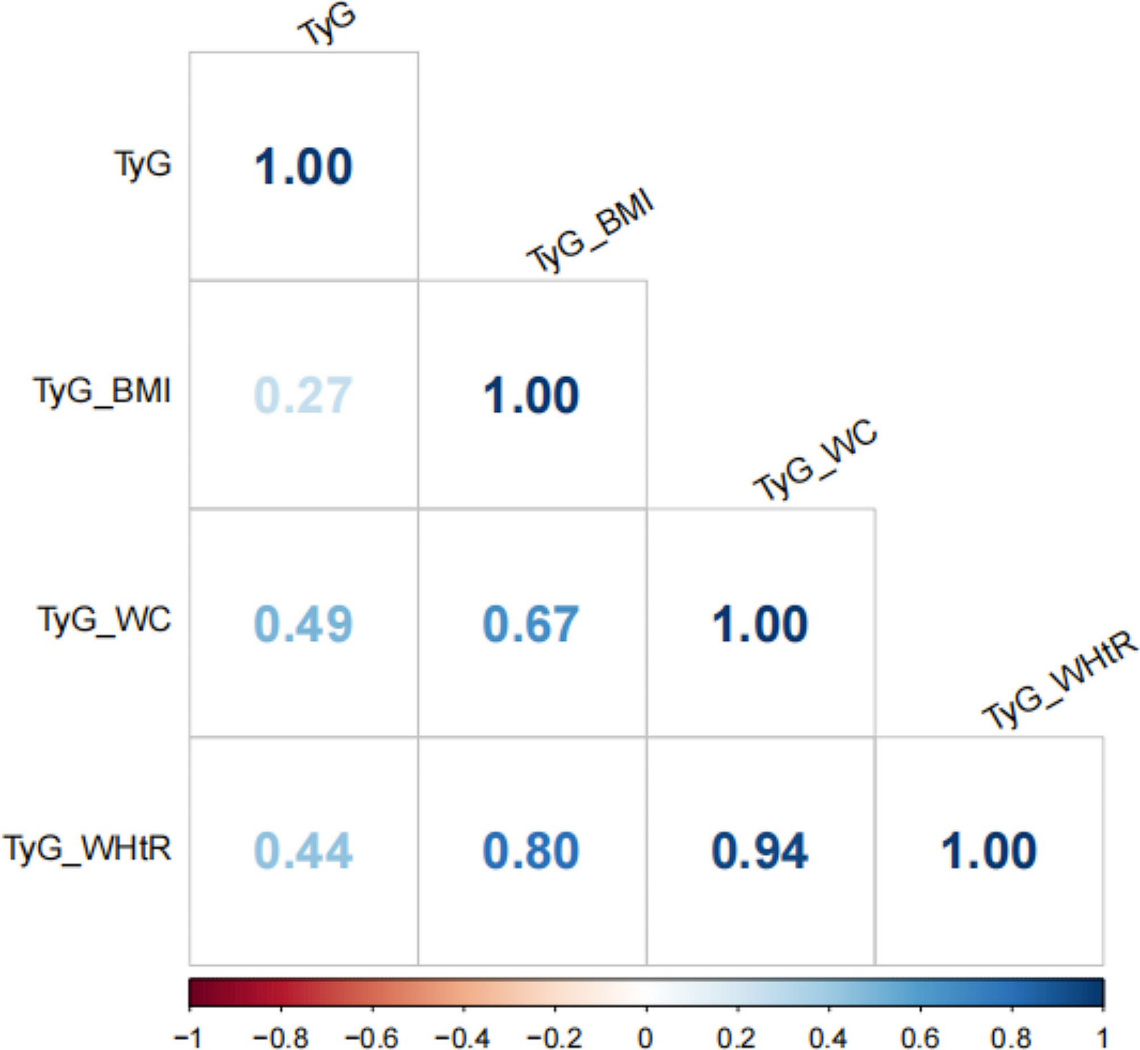
Spearman’s correlation of TyG and modified indices

A total of 7 115 participants (3894 men and 3221 women) were included in this study. The mean (SD) age was 58.8 (9.0) years. Baseline characteristics according the TyG tertile groups are shown in Table 1. People in the highest tertile of TyG index were more likely to be male (< 0.001) and having diabetes (*P* < 0.001). During a mean follow-up of 7.0 years, 2136 participants developed cardiovascular disease, including 1633 cases of coronary heart disease and 719 cases of stroke. There is no significant difference of BMI level (*P* = 0.898). Figure 1 presents the Spearman’s coefficients of paired correlation among TyG, TyG-BMI, TyG-WC, and TyG-WHtR. TyG was not strongly correlated with the modified TyG indices, and the Spearman’s coefficients for TyG-BMI, TyG-WC, and TyG-WHtR were 0.27, 0.49, and 0.44, respectively.

**Table 1:** Characteristics of 7 115 participants according to baseline TyG levels

	Overall	Baseline TyG index		
		Tertile 1	Tertile 2	Tertile 3
Participants, No.	7115	2372	2372	2371
Age, years, mean (SD)	58.77 (8.96)	58.74 (9.35)	59.00 (8.83)	58.56 (8.70)
Sex, Female, n (%)	3221 (45.3)	1242 (52.4)	1043 (44.0)	936 (39.5)
Residence, n (%)				
Rural	5857 (82.4)	2026 (85.5)	1955 (82.5)	1876 (79.2)
Urban	1252 (17.6)	344 (14.5)	416 (17.5)	492 (20.8)
Marriage, married, n (%)	6340 (89.1)	2103 (88.7)	2111 (89.0)	2126 (89.7)
Educational level, n (%)				
Primary	4901 (68.9)	1631 (68.8)	1677 (70.7)	1593 (67.2)
Secondary	1475 (20.7)	491 (20.7)	460 (19.4)	524 (22.1)
Third	739 (10.4)	250 (10.5)	235 ( 9.9)	254 (10.7)
Smoking status, n (%)				
Never	4244 (59.7)	1409 (59.5)	1401 (59.1)	1434 (60.6)
Former	503 ( 7.1)	151 ( 6.4)	166 ( 7.0)	186 ( 7.9)
Current	2360 (33.2)	810 (34.2)	802 (33.9)	748 (31.6)
Current drinking, n (%)	2449 (34.5)	833 (35.2)	817 (34.5)	799 (33.7)
BMI ^a^, kg/m^2^				
Continuous	23.62 (5.28)	23.61 (5.27)	23.59 (5.27)	23.66 (5.30)
<23.9	3998 (56.2)	1353 (57.0)	1318 (55.6)	1327 (56.0)
24-27.9	1554 (21.8)	489 (20.6)	548 (23.1)	517 (21.8)
≥28	1563 (22.0)	530 (22.3)	506 (21.3)	527 (22.2)
Hypertension, n (%)	3307 (46.5)	1086 (45.8)	1124 (47.4)	1097 (46.3)
Diabetes, n (%)	1285 (18.1)	174 ( 7.3)	316 (13.3)	795 (33.5)

### Relationships of TyG and modified TyG indices with cardiovascular disease

**Fig. 2:**
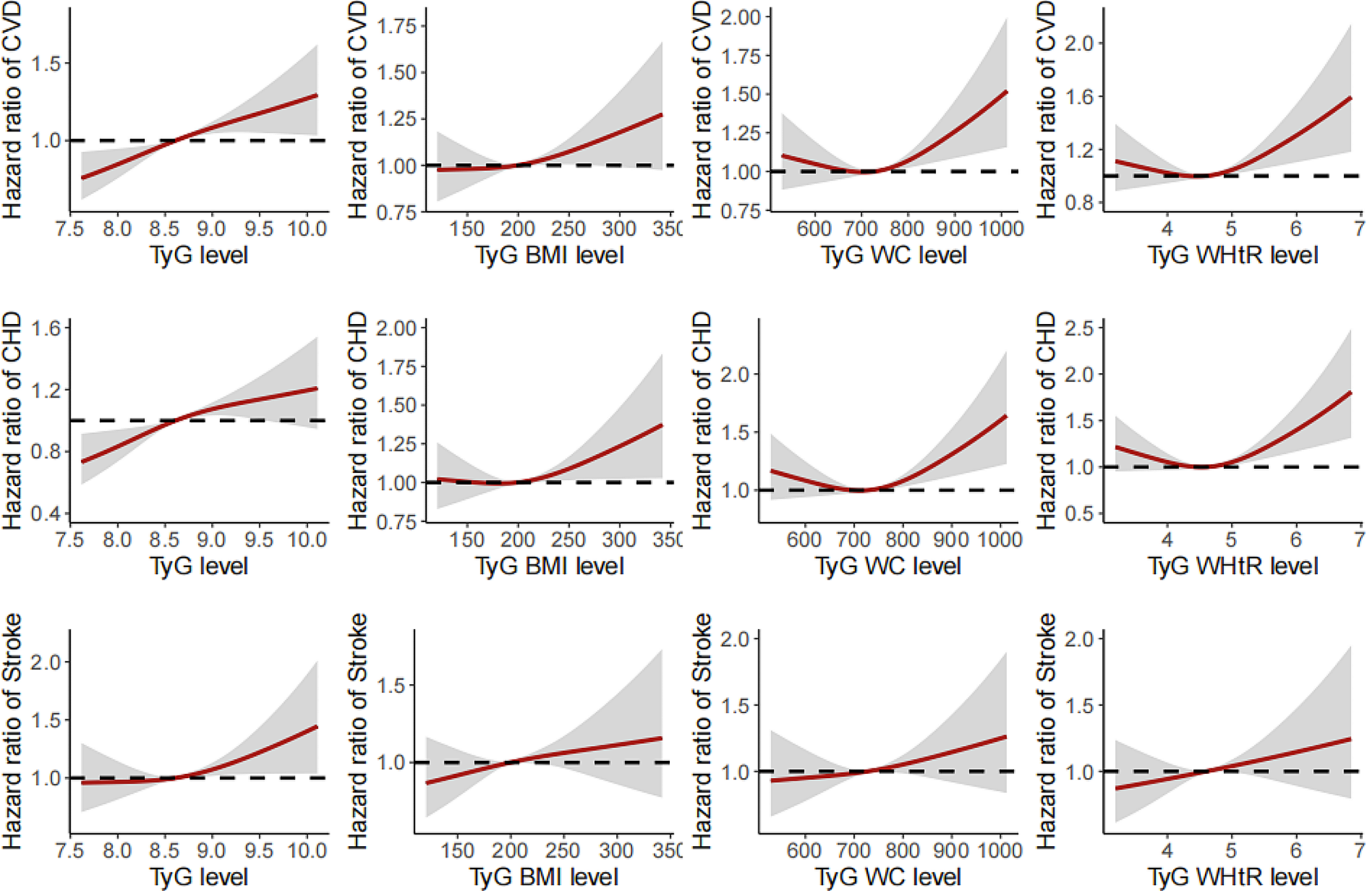
Dose-responsive relationship of the TyG and modified indices with the risk of cardiovascular diseases, coronary heart disease and stroke

Figure 2 presents the positive dose-responsive relationships of TyG and modified TyG indices with the risk of cardiovascular disease, coronary heart disease and stroke. The Kaplan–Meier curves of the cumulative incidence of cardiovascular disease, coronary heart diseases and stroke were shown in Figure S1-Figure S3. In the fully adjusted model (Table 2), when compared with people in the lowest tertile, those in the highest tertile of TyG and TyG-WHtR were significantly associated with a higher risk of new-onset cardiovascular disease, and the adjusted HR (95% CI) were 1.215 (1.088–1.356) and 1.112 (1.002–1.235), respectively. TyG-BMI and TyG-WC were not significantly associated with the incidence of cardiovascular disease, and the adjusted HR (95% CI) were 1.073 (0.967–1.191) and 1.078 (0.970–1.198). Table 3 shows the associations between TyG and modified TyG indices in terms of new-onset coronary heart disease and stroke. TyG index, but not TyG-BMI, TyG-WC and TyG-WHtR, was associated with a higher risk of coronary heart disease (T3 vs. T1: HR, 1.200; 95 CI, 1.057–1.362). TyG, TyG-BMI and TyG-WHtR, but not TyG-WC, were significantly associated with the incidence of stroke, and the adjusted HR (95% CI) were 1.249 (1.039–1.501), 1.200 (1.001–1.438), and 1.236 (1.031–1.483), respectively.

**Table 2:** Associations of TyG index and modified indices with cardiovascular disease onset

Index	Tertile 1	Tertile 2		Tertile 3	
		HR (95% CI)	*P* value	HR (95% CI)	*P* value
TyG					
Model 1	Ref	1.101(0.989–1.225)	0.078	1.252(1.127–1.39)	< 0.001
Model 2	Ref	1.091(0.979–1.214)	0.114	1.215(1.088–1.356)	0.001
TyG BMI					
Model 1	Ref	1.022(0.92–1.135)	0.687	1.08(0.974–1.199)	0.144
Model 2	Ref	1.014(0.913–1.127)	0.793	1.073(0.967–1.191)	0.184
TyG WC					
Model 1	Ref	0.999(0.899–1.109)	0.982	1.088(0.981–1.207)	0.109
Model 2	Ref	0.997(0.897–1.108)	0.951	1.078(0.970–1.198)	0.162
TyG WHtR					
Model 1	Ref	1.007(0.906–1.12)	0.891	1.125(1.014–1.247)	0.026
Model 2	Ref	1.005(0.904–1.117)	0.928	1.112(1.002–1.235)	0.046

Abbreviations: HR, hazard ratio; CI, confidence interval; BMI, body mass index; TyG, triglyceride-glucose index; WC: waist circumference; WHtR: waist-to‐height ratio

Model 1: age and sex were adjusted; model 2: age, sex, residence, marriage, education level, smoking status, current drinking, hypertension, and diabetes were adjusted

**Table 3:** Associations of TyG index and modified indices with onset of coronary heary disease and stroke

	HR (95% CI)			
	TyG	TyG BMI	TyG WC	TyG WHtR
*CHD*				
Model 1				
Tertile 1	Ref			
Tertile 2	1.158(1.025–1.309)	1.031(0.914–1.162)	0.948(0.84–1.07)	0.96(0.851–1.084)
Tertile 3	1.251(1.108–1.412)	1.079(0.958–1.215)	1.095(0.974–1.231)	1.121(0.997–1.261)
Model 2				
Tertile 1	Ref			
Tertile 2	1.141(1.009–1.29)	1.015(0.9-1.145)	0.942(0.834–1.064)	0.952(0.843–1.075)
Tertile 3	1.2(1.057–1.362)	1.066(0.946–1.201)	1.08(0.958–1.217)	1.103(0.979–1.242)
*Stroke*				
Model 1				
Tertile 1	Ref			
Tertile 2	0.9(0.747–1.086)	1.081(0.9-1.298)	1.154(0.963–1.383)	1.082(0.9–1.3)
Tertile 3	1.258(1.056–1.499)	1.199(1.002–1.435)	1.155(0.963–1.386)	1.24(1.037–1.484)
Model 2				
Tertile 1	Ref			
Tertile 2	0.902(0.747–1.089)	1.091(0.908–1.312)	1.156(0.964–1.387)	1.094(0.91–1.316)
Tertile 3	1.249(1.039–1.501)	1.2(1.001–1.438)	1.147(0.954–1.381)	1.236(1.031–1.483)

Abbreviations: HR, hazard ratio; CI, confidence interval; BMI, body mass index; TyG, triglyceride-glucose index; WC: waist circumference; WHtR: waist-to‐height ratio; CHD: coronary heart disease

Model 1: age and sex were adjusted; model 2: age, sex, residence, marriage, education level, smoking status, current drinking, hypertension and diabetes were adjusted

### Predictive capacity comparison

**Fig. 3:**
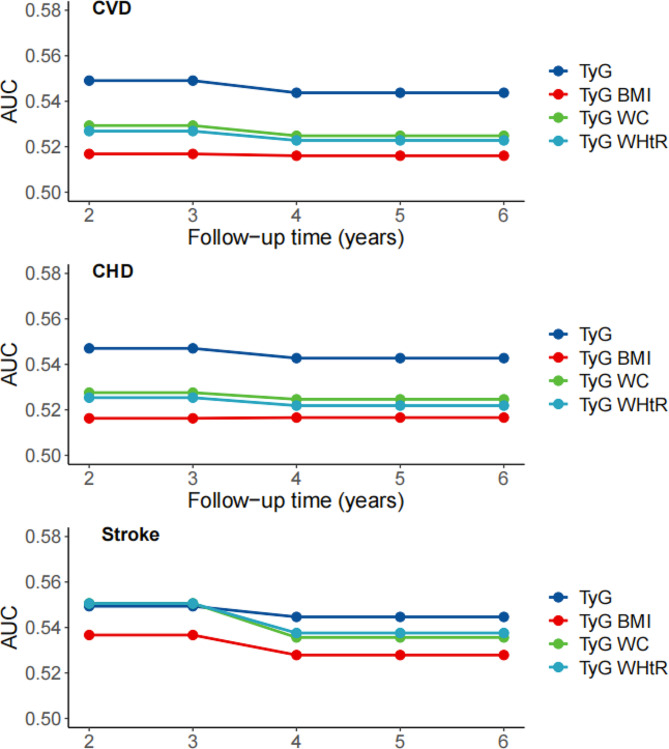
Time-dependent predictive capacity of TyG and modified indices for cardiovascular diseases, coronary heart disease and stroke

We calculated the time-dependent Harrell’s C-indices of TyG, TyG-BMI, TyG-WC, and TyG-WHtR that were significantly related to new-onset cardiovascular disease, coronary heart diseases and stroke (Fig. 3). The overall C-index values were 0.544 (95% CI: 0.528–0.560) for TyG, followed by TyG-WC of 0.525 (95% CI: 0.508–0.541), TyG-WHtR of 0.523 (95% CI: 0.506–0.539), and TyG-BMI of 0.516 (95% CI: 0.503–0.532) in terms of new-onset cardiovascular disease. Similarly, the C-index values were 0.543 (95% CI: 0.525–0.560) for TyG, followed by TyG-WC of 0.525 (95% CI: 0.507–0.542), TyG-WHtR of 0.522 (95% CI: 0.504–0.540), and TyG-BMI of 0.517 (95% CI: 0.502–0.534) in terms of new-onset coronary heart disease, and 0.545 (95% CI: 0.517–0.572) for TyG, followed by TyG-WHtR of 0.538 (95% CI: 0.510–0.565), TyG-WC of 0.536 (95% CI: 0.508–0.563), and TyG-BMI of 0.528 (95% CI: 0.501–0.555) in terms of new-onset stroke. In addition, TyG index shows the highest C-index of cardiovascular diseases both in men and women (Figure S4).

## Discussion

In this national longitudinal cohort study based on the Chinese health examination population, we comprehensively investigated the associations between TyG index and modified TyG indices with new-onset cardiovascular disease and compared the time-dependent predictive capacity. Overall, TyG index is moderately correlated with other modified TyG indices. TyG index and TyG-WHtR, but not TyG-BMI nor TyG-WC, are independently predictors of future cardiovascular disease onset. Of note, TyG index has the highest predictive capacity for cardiovascular risk.

Insulin resistance plays a key role in atherosclerosis and cardiovascular disease, recognized by mechanism research, epidemiology study and genetic data. One mechanism by which insulin resistance leads to atherosclerosis and cardiovascular disease is via vascular stiffness, and defective insulin signaling in atherosclerotic lesion cells also links insulin resistance and atherosclerotic vascular disease [17, 18]. Genetically instrumented insulin resistance is causally associated with cardiovascular risk, such as heart failure [19] and ischemic stroke [20]. In epidemiological studies, the association between insulin resistance estimated by HOMA-IR has been confirmed [21].

Given the data of insulin levels are not widely measured, there are several surrogate markers of insulin resistance, of which TyG index is a reliable indicator. Previous studies have recognized the relationship between TyG index and cardiometabolic outcomes, including hypertension [11], diabetes [22], and cardiovascular diseases [23]. In addition to the effects of metabolic biomarkers like triglyceride and glucose, the content and distribution of body fat are closed correlated with insulin resistance. Hormones and cytokines from adipocytes can enhance or inhibit both glycemic response and insulin signaling [24]. Taking account for the crosstalk between insulin resistance and obesity, an increasing number of studies have been investigating whether combing TyG index and obesity-related parameters, such as BMI, WC and WHtR could enhance the risk stratification of cardiometabolic outcomes. In our analysis, we found that TyG index is moderately correlated with the modified TyG indices, of which the correlation coefficients range from 0.27 to 0.49 (Fig. 1). But the correlation coefficients among TyG-BMI, TyG-WC and TyG-WHtR range from 0.67 to 0.94, which means TyG index capture the distinct aspect compared to the modified TyG indices.

The results in terms of modified TyG indices including TyG-BMI, TyG-WC and TyG-WHtR are highly controversial given different disease outcomes and study design. In a cross-sectional survey of an elderly population, Ke et al. found that combining the TyG index with BMI, WC, and WHtR could not further improve the performance of TyG index in recognizing the risk of diabetes [25]. Zhang et al. found that TyG-WC was more strongly related with the prevalence of prediabetes and diabetes, which had the highest prediction accuracy [9]. Kuang et al. found that TyG index combined with BMI, WC, and WHtR can improve the prediction of the risk of diabetes using a cohort design [10]. Li et al. indicated that TyG-WHtR and TyG-BMI outperformed the TyG index alone to predict the new-onset of diabetes [26]. Lee et al. reported the sex-specific relationship between modified TyG indices and the incidence of hypertension [11]. Another cohort study found that TyG-related markers combining obesity parameters are superior to identify metabolic syndrome in both genders [27].

Given that evidence is inconsistent about the predictive capacity of the TyG index and modified TyG indices on cardiovascular outcomes, our study evaluated the association between TyG index and the modified TyG indices with the new-onset cardiovascular diseases using a Chinese national cohort. We found that TyG index along is most strongly associated with the incidence of coronary heart disease, stroke, and the composite outcome. TyG index also outperformed other modified TyG indices in predicting the future cardiovascular risk (Fig. 3), and the C-index was largely consistent from previous study among other population [28]. WC is a useful assessment parameter for abdominal obesity, which is closely associated insulin resistance [29]. WHtR is another a useful tool for assessing central obesity after adjusting for statures [30, 31]. However, the findings did not support the superior role of modified TyG indices for predicting the onset of cardiovascular events. In addition, a previous study indicated that TyG index had a higher predictive power for cardiovascular disease than HOMA-IR [32]. Our findings indicated that there is a J-shaped relationship between modified TyG indices and new-onset coronary heart disease, while Dang et al. reported a potential linear relationship [33]. The distinct study designs of cohort and cross-sectional frameworks could partially explain the difference. In addition, participants aged 45 years and older were included in our study, and the distinct age distribution could be another reason. On the other hand, Park et al. reported that TyG-WC and TyG-WHtR had better predictive performances for new-onset cardiovascular disease than TyG and TyG-BMI only in participants without diabetes [34]. Given the current analysis, findings supported the superiority of TyG index over the modified TyG indices using obesity related parameters for the risk stratification and prediction of future cardiovascular disease in clinical practice among middle-aged and older Chinese adults. Further studies are needed to reveal the distinct effects of TyG index and modified TyG indices on cardiovascular health among different population and subgroups, such as diabetes status [23].

Indeed, the specific reasons for the differential relationship between the TyG index and modified TyG indices with cardiovascular disease are not clear. There are several possible explanations. TyG index, derived from glucose and triglyceride, captures the status of glucose metabolism and hyperlipidemia, which determine the risk of atherosclerosis diseases than body weight and visceral fat [35]. TyG index itself is a comprehensive indicator of adipose volume, density, and distribution [36]. In addition, CHARLS cohort recruited people aged above 45 years. Hyperglycemia and hyperlipidemia could play a more important role in further incidence of cardiovascular disease among the middle-aged and elder population than obesity parameters. Besides, weight loss could be associated with an increase in all-cause and cause-specific mortality in the healthy older adults [37]. Moreover, the effects of TyG index and and modified TyG indices are possibly distinct between low- or middle-income countries and high-income countries, which is suggested by the PURE study [23].

### Limitation

Several limitations of the current study should be acknowledged. First, owing to the observational study design, we could not confer the causal association between TyG index and modified TyG indices with cardiovascular disease. Further genetic studies and clinical trials are needed to validate the evidence strengthen. Second, the data of cardiovascular disease onset is self-reported; however, it has been shown the high consistency between self-reported disease diagnosis and medical records in terms of cardiovascular event [38]. Third, there is a possibility of residual or unmeasured confounding bias. Further studies are needed to validate the findings in other populations, not limited to the middled-aged and older.

## Conclusions

TyG index and TyG-WHtR were significantly associated with new-onset cardiovascular disease among the middle-aged and older Chinese adults. The findings supported the superiority of TyG index over the modified TyG indices using obesity related parameters for the risk stratification and prediction of future cardiovascular disease.

### Electronic supplementary material

Below is the link to the electronic supplementary material.

Supplementary Material 1: **Additional File: Fig. S1-S4**. **Fig. S1** [K‒M plot of cardiovascular diseases by the tertile groups of TyG and modified indices]. **Fig. S2** [K‒M plot of coronary heart diseases by the tertile groups of TyG and modified indices]. **Fig. S3** [K‒M plot of stroke by the tertile groups of TyG and modified indices]. **Fig. S4** [Sex-specific time-dependent predictive capacity of TyG and modified indices for cardiovascular diseases, coronary heart disease and stroke]

## Data Availability

The data sets used and/or analyzed during the current study are publicly available or from the corresponding author upon reasonable request.

## Declarations

### Originality of content

All authors verify that all information and materials in the manuscript are original.
