# Supplementary material for: Comparison of triglyceride glucose index and modified triglyceride glucose indices in prediction of cardiovascular diseases in middle aged and older Chinese adults
Source: Cardiovasc Diabetol. 2024 May 29;23:185. doi: 10.1186/s12933-024-02278-z (PMC11138075; doi:10.1186/s12933-024-02278-z)
Supplement: Supplementary file 1 — Supplementary Material 1: Additional File: Fig. S1-S4. Fig. S1 [K‒M plot of cardiovascular diseases by the tertile groups of TyG and modified indices]. Fig. S2 [K‒M plot of coronary heart diseases by the tertile groups of TyG and modified indices]. Fig. S3 [K‒M plot of stroke by the tertile groups of TyG and modified indices]. Fig. S4 [Sex-specific time-dependent predictive capacity of TyG and modified indices for cardiovascular diseases, coronary heart disease and stroke] [file 12933_2024_2278_MOESM1_ESM.pdf]

Additional File

Comparison Between Triglyceride Glucose Index and Modified Indices to Predict New-Onset Cardiovascular Disease in Middle-Aged and Older Adults

Cancan et. al,

Fig. S1-S4. **Fig. S1** [K–M plot of cardiovascular diseases by the tertile groups of TyG and modified indices]. **Fig. S2** [K–M plot of coronary heart diseases by the tertile groups of TyG and modified indices]. **Fig. S3** [K–M plot of stroke by the tertile groups of TyG and modified indices]. **Fig. S4** [Sex-specific time-dependent predictive capacity of TyG and modified indices for cardiovascular diseases, coronary heart disease and stroke]

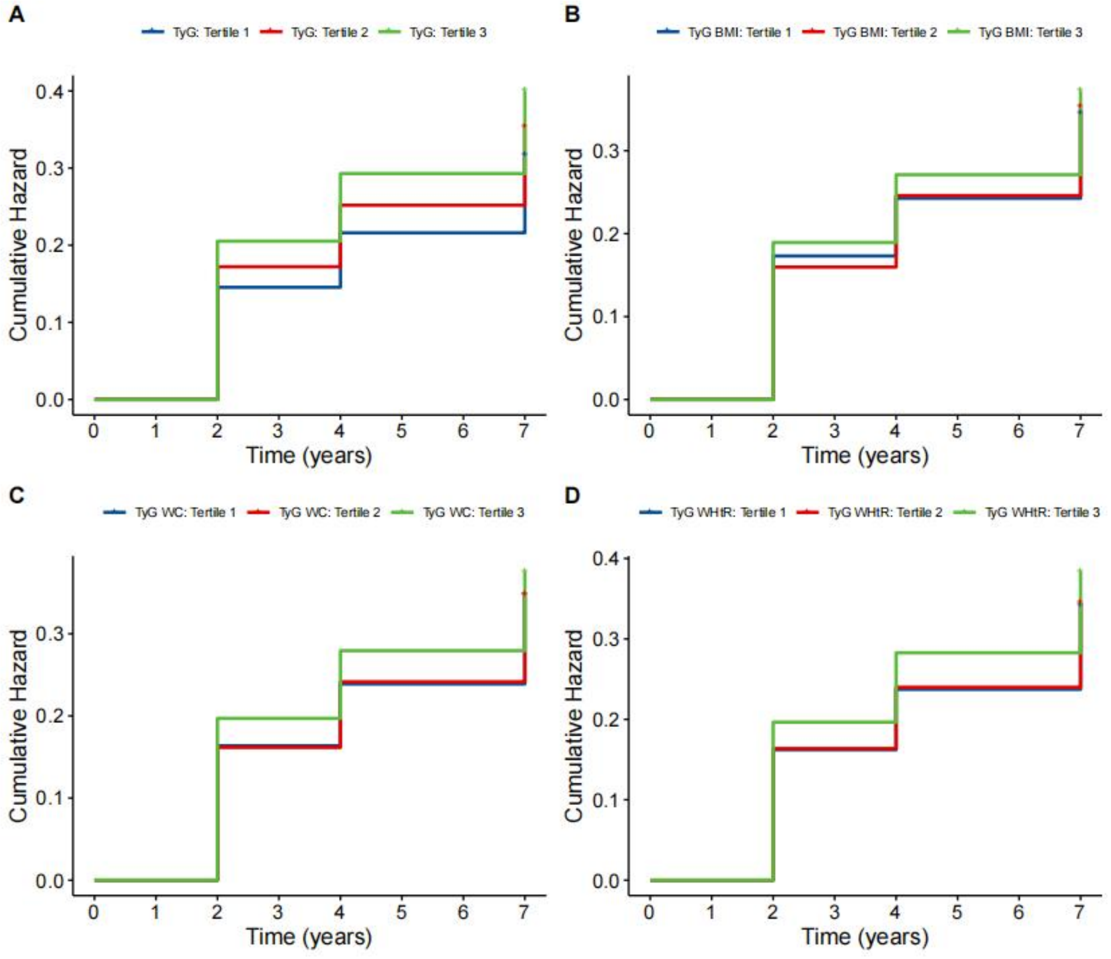

Fig. S1 [K–M plot of cardiovascular diseases by the tertile groups of TyG and modified indices].

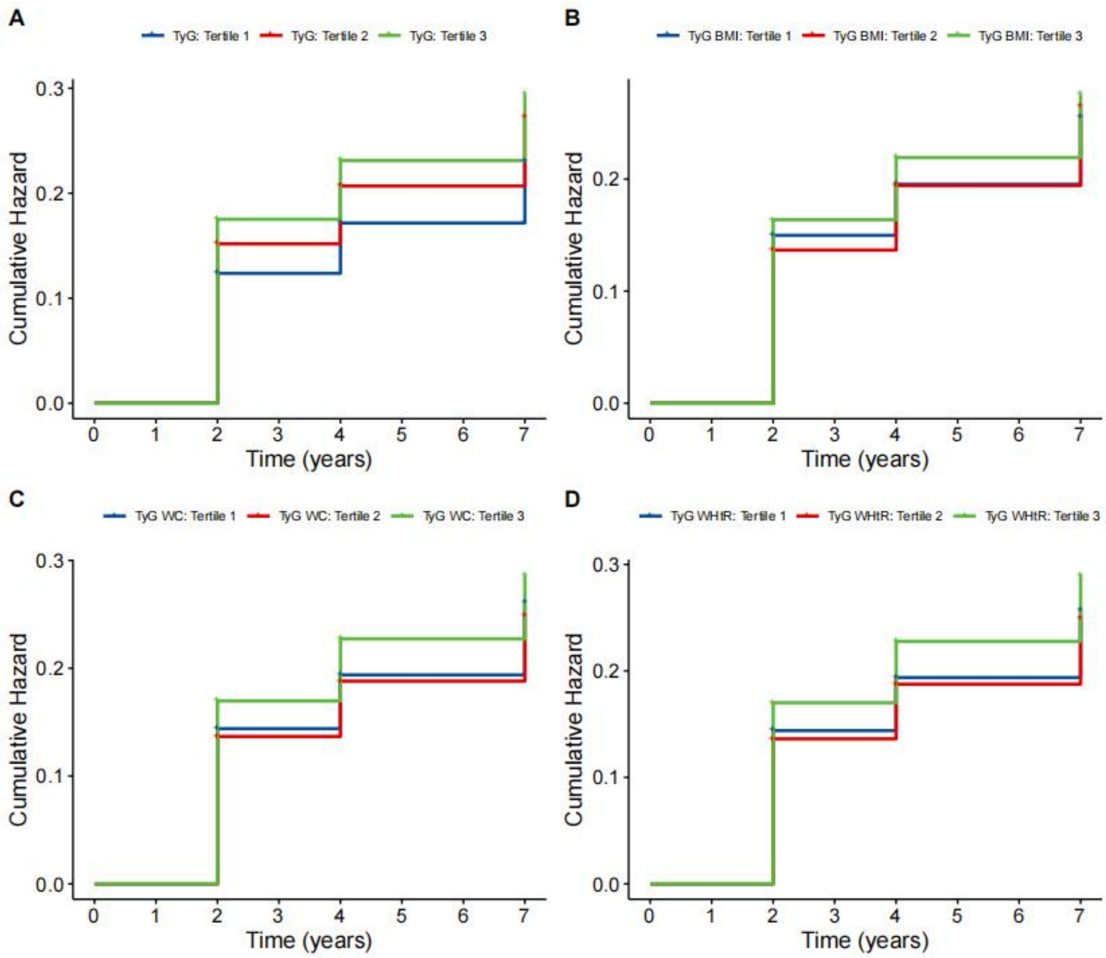

Fig. S2 [K–M plot of coronary heart diseases by the tertile groups of TyG and modified indices].

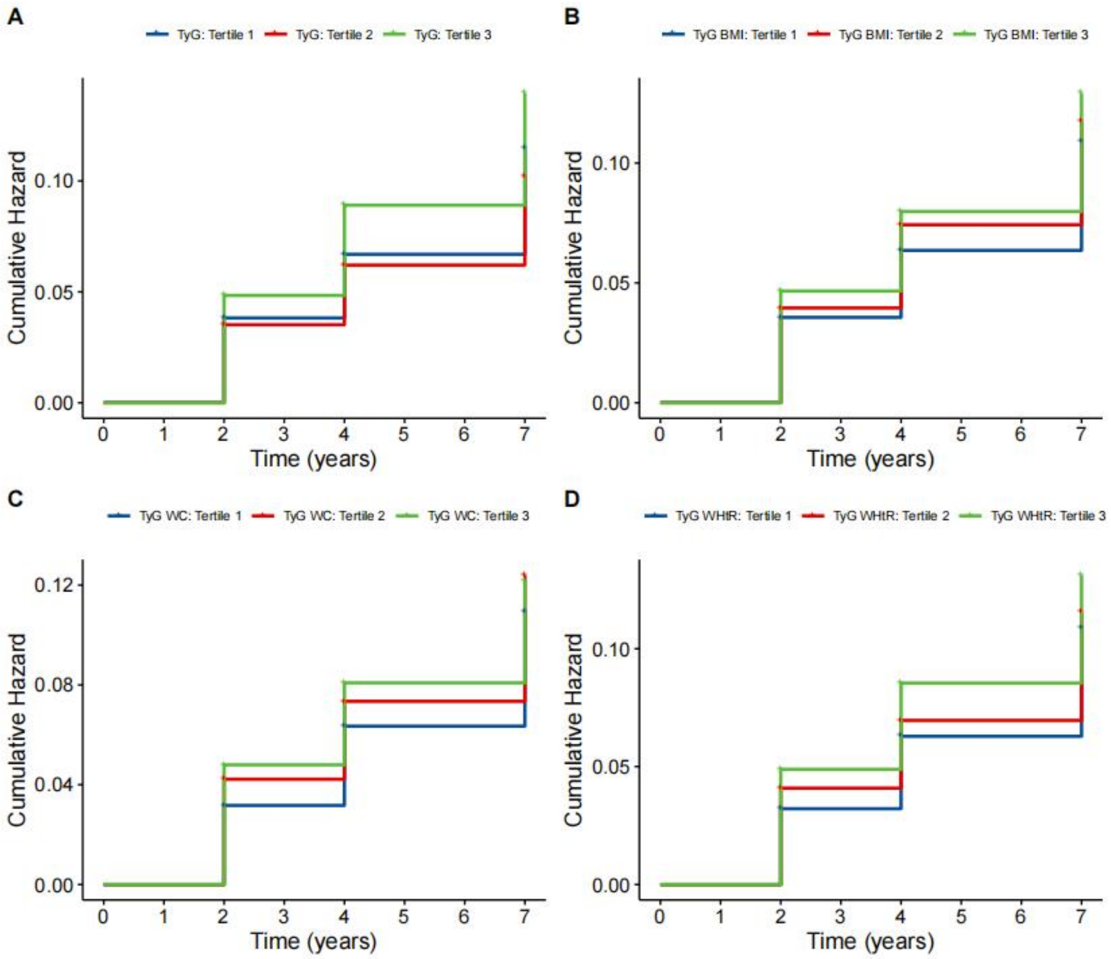

Fig. S3 [K–M plot of stroke by the tertile groups of TyG and modified indices].

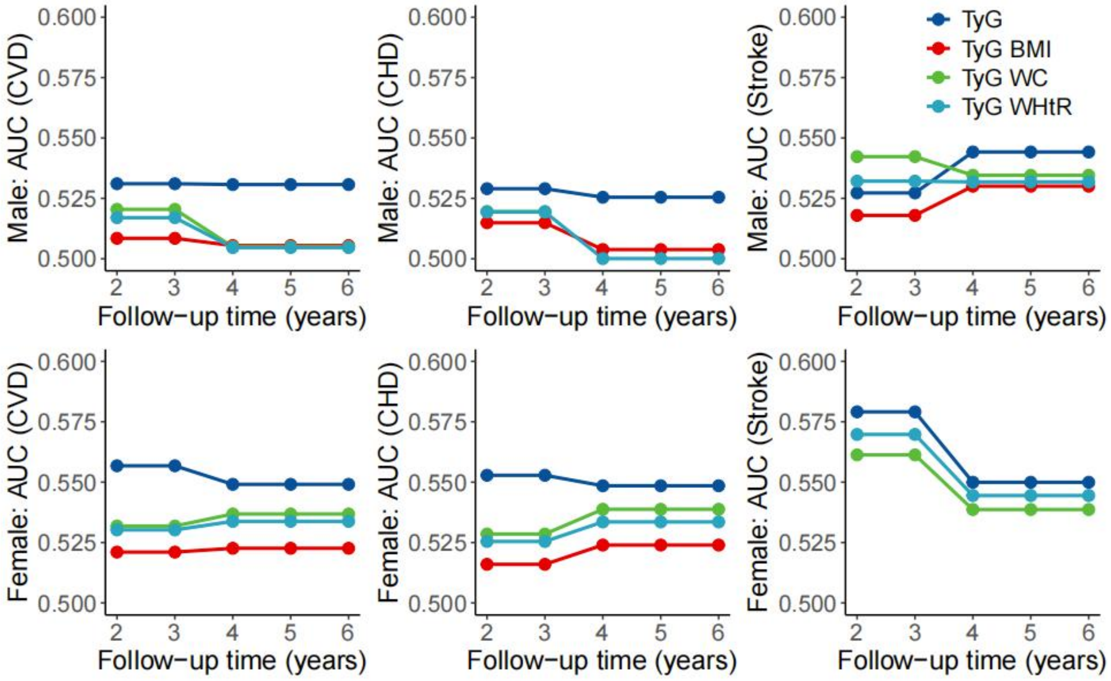

Fig. S4 [Sex-specific time-dependent predictive capacity of TyG and modified indices for cardiovascular diseases, coronary heart disease and stroke]
